# Supplementary material for: Effects of urbanization and land expropriation on the livelihoods of peri-urban farmers in North Wollo Zone, Ethiopia
Source: PLoS One. 2025 Jun 26;20(6):e0325985. doi: 10.1371/journal.pone.0325985 (PMC12200778; doi:10.1371/journal.pone.0325985)
Supplement: Attachment — Submitted filename: Land Expropriation_Reviewer Comments.docx [file pone.0325985.s001.docx]

**Land Expropriation, Compensation, and Livelihood Diversification: Insights from Peri-**

**Urban Farmers in Woldia city and selected Towns of North Wollo Zone, Ethiopia**

**Comments**

I enjoyed reading this paper. The authors can consider the following comments to improve their work:

- Consider providing a more concise historical context of urbanization's impact specifically in the study area to ground the research in a specific socio-economic context.
- Consider adding an area of study map
- Clarify and elaborate on the unique characteristics of peri-urban farming in Woldia and selected towns compared to other regions in Ethiopia, as this can strengthen the argument about the significance of studying this specific area.
- I find the paper lacking a robust literature review, conceptual or theoretical section (this could focus on 1. global and regional trends in urbanization, especially in developing countries, highlighting how urban expansion affects peri-urban lands and communities, 2. theories related to livelihood diversification and compensation, such as the Sustainable Livelihood Framework, to understand the strategies households employ in response to land loss, 3. Review empirical studies that document the social, economic, and psychological effects of land expropriation on peri-urban farmers, focusing on both challenges and adaptive strategies. 4. Analyzing the specific legal and policy frameworks governing land expropriation in Ethiopia, noting recent changes and their implications for land compensation and livelihood restoration. 5. Identifying gaps in existing literature, particularly concerning long-term impacts on livelihoods and the effectiveness of compensation measures, justifying the need for the current study’s focus.
- The sample size and sampling technique are well-explained, but the paper could benefit from a more detailed discussion on the limitations of the chosen methods, especially concerning potential biases in key informant interviews and focus group discussions.
- Consider discussing the validation of the structured questionnaires used for collecting quantitative data to enhance credibility.
- While the use of binary logistic regression is appropriate, ensure the model assumptions and the choice of variables are justified in more detail. This can include discussing why certain variables were included or excluded and the potential impact of this on the findings.
- Provide a more critical analysis of the quantitative data. For instance, discussing the implications of high inflation and lack of urban living skills on livelihood diversification could add depth.
- Results are comprehensive but could be better organized to lead the reader through the findings more clearly. Consider using subheadings to distinguish between different aspects of the results, such as economic impacts versus social impacts.
- A separate strong discussion might be good for this paper (Discuss how the findings align with or differ from theoretical expectations related to livelihood diversification and compensation, Compare the results with similar studies in other regions or contexts, highlighting unique findings and common trends., Based on the findings, recommend specific policy changes or interventions that could improve compensation practices and support livelihood diversification for affected farmers., Discuss practical measures that local governments and development agencies can implement to mitigate the negative impacts of land expropriation on peri-urban farmers.
- The policy implications are practical; however, they could be strengthened by suggesting specific policy measures or programs, detailing how they could be implemented within the current Ethiopian administrative and legal framework.
- Consider adding future research directions, suggesting specific areas that need further investigation based on the study's findings.
- Proof read the paper
