# Supplementary material for: Effects of urbanization and land expropriation on the livelihoods of peri-urban farmers in North Wollo Zone, Ethiopia
Source: PLoS One. 2025 Jun 26;20(6):e0325985. doi: 10.1371/journal.pone.0325985 (PMC12200778; doi:10.1371/journal.pone.0325985)
Supplement: Attachment — Submitted filename: Response to Reviewers.docx [file pone.0325985.s004.docx]

**Response to Reviewers**

**Manuscript PONE-D-25-01172**

**Title**: *Land Expropriation, Compensation, and Livelihood Diversification: Insights from Peri-Urban Farmers in Woldia City and Selected Towns of North Wollo Zone, Ethiopia*

We would like to thank the editor and reviewers for their valuable feedback and suggestions on our manuscript. We have carefully revised the manuscript in response to the comments provided, and we believe these revisions have substantially improved the quality of the paper. Below, we outline our responses to each of the reviewers' comments.

**General Responses:**

We appreciate the positive feedback regarding the relevance of our study and its focus on urban expansion and land expropriation in Ethiopia, especially in peri-urban areas. We have made significant revisions to address the concerns raised by the reviewers. Specifically, we have clarified the methodology, refined the title to be more specific, and provided more detailed explanations regarding the methods, variables, and theoretical frameworks used in the study.

**Editor comment #:**

**Comment 1: Clarify the methodology strategy. It is not clear where the secondary data is incorporated in the analysis.**

Response: Thank you for your comment. We have revised and clarified in the methodology section, and due to data limitations, we used only primary data sources for this study.

**Comment 2: Detail the variables that entail secondary data from the institutional reports related to land expropriation and compensation.**

Response: Thank you for your comment. We have revised and clarified in the methodology section, and due to data limitations, we used only primary data sources for this study.

**Comment 3: Clarify methodology and interpretation of findings.**

Response: We have refined the explanation of our methodology and findings. We now provide a clearer interpretation of both the quantitative and qualitative results in this version of the paper.

**Reviewer #1 comments:**

1. **Title Inadequacy & Lack of Insights:** The title promises "insights," but the manuscript mostly presents results with limited analysis or in-depth discussion. The findings should be linked to established theory and policy implications, specifically the insights that the title promises. The title needs to reflect the limitations of the work as well (e.g., "Livelihood Diversification Strategies") and may benefit from being more specific (e.g., "A Mixed-Methods Analysis").

Response: Thank you for this valuable comment. We have revised the title to more accurately reflect the study’s focus on livelihood diversification strategies following land expropriation. The new title is more specific and aligned with the content of the manuscript.

2. **Mixed Methods Inconsistency:** The manuscript claims a mixed methods approach, but the discussion heavily focuses on quantitative findings. The qualitative data and their themes are not explicitly presented. This makes the approach feel more quantitative and misses key opportunities for deeper insights, which requires a major re-working of the manuscript.

Response: We have addressed the concern regarding the mixed methods approach. In the revised manuscript, we now clearly explain the integration of qualitative and quantitative data, including how the qualitative interviews and thematic analysis support and refine the quantitative findings.

3. **Unclear Justification for Mixed Methods:** The introduction mentions "mixed methods" but does not explain why this approach was chosen, and why that is essential. The authors must explain how the qual/quant approaches complement each other. (For example, do qualitative data refine the quant results?) This must be done in the methods and introduced in the research design section.

Response: In response to this comment, we have added a section in the methodology that justifies the use of mixed methods, explaining how qualitative data complements quantitative results, particularly in understanding the complexity of livelihood diversification strategies.

4. **Missing Qualitative Analysis:** The method mentions thematic analysis, yet there is no explicit discussion of the themes or how they were identified. Quotes or examples from interviews and focus groups should be included to ground the discussion. This is a major shortcoming that must be corrected.

Response: We have now included a detailed discussion of the themes from the qualitative interviews and focus group discussions. Relevant quotes from interviews and discussants have been added to provide richer context and support our findings.

5. **Research Design Vague:** The paper lacks a clear description of the study design. The manuscript needs to articulate:
   o The specific mixed methods design (e.g., convergent parallel, explanatory sequential).
   o How the quantitative and qualitative data were integrated (or not, in this case).

Response: We have clarified the research design, specifying that the study follows an explanatory sequential mixed methods design. We now describe how quantitative and qualitative data were collected and integrated.

6. **Problem Statement and Gap:** The problem statement should clearly explain the existing research and knowledge gap that this study fills. The gap is not clear in the manuscript.

Response: We have revised the problem statement to more clearly articulate the gap in existing research.

7. **Statistical Rigor:** There is minimal discussion of the statistical methods beyond mentioning "binary logistic regression." There is no explanation as to why the model variables were chosen, or if any model specification tests were run.

Response: We have elaborated on the statistical methods used, including the rationale for choosing binary logistic regression and the process of selecting model variables. We also describe any model specification tests that were performed.

8. **Affiliation Format:** As noted, affiliations are overly detailed. Simply state the department or faculty, and the institution, for example, Department of Economics, Woldia University.

Response: We have revised the affiliations to match the required format, stating the department and institution clearly.

9. **Abstract Weakness:** The abstract lacks specificity, the gap is not clear, and does not summarize the key findings. This needs to be revised, making sure that the methods used are clearly stated and the summary points of the results are included.

Response: We have revised the abstract accordingly.

10. **Study Area Maps:** Including a map to visualize the study area is important and a suggestion that the authors should strongly consider. This will help readers understand the geographical context.

Response: As suggested, we have included a map of the study area to help visualize the geographical context.

11. **Overuse of "Peri-Urban":** The term "peri-urban" is frequently used. It is ok, but should be better described as to how this study area fits into the definition of a peri-urban environment, and if other studies define the study area as such.

Response: We have revised the manuscript to reduce redundancy in the use of the term "peri-urban." Additionally, we have clarified how the study area fits into the definition of peri-urban environments and compared it to other regions in Ethiopia in the literature section.

12. **Table Clarity:** Tables could be improved (i.e., table 2), which need better headings and subheadings for better readability and interpretation. Tables should have a clear and concise explanation or interpretation of the results in the text.

Response: We have revised the tables, including improving headings and subheadings for clarity and readability. Additionally, each table now has a clearer explanation in the text.

13. **Variable Interpretation:** the variables in table 1 need to be described a bit better. The reader cannot intuit all the meanings of all the variables without explanation.

Response: We have added more detailed descriptions of the variables in Table 1, making it easier for readers to understand their meanings and significance.

14. **Ethical Concerns:** Ethical Concerns: The paper mentions ethical approval and informed consent but should include more detail (e.g., if participants were told that participation was voluntary or that their data could be shared anonymously).

Response: We have added more detail to the ethical section, including information on voluntary participation, informed consent, and the anonymization of participant data.

**Reviewer #2:**

**Comment 1: Consider providing a more concise historical context of urbanization’s impact specifically in the study area to ground the research in a specific socio-economic context.**

Response: Thank you for your comment. We have added a more concise historical context regarding urbanization’s impact on the study area to better ground the research in the local socio-economic environment.

**Comment 2: Consider adding an area of study map.**

Response: As per your suggestion, we have added a map to the manuscript.

**Comment 3: Clarify peri-urban farming in Woldia and selected towns compared to other regions in Ethiopia.** Response: We have expanded the manuscript to include a more detailed description of peri-urban farming in the study area in this version of the paper.

**Comment 4: Lack of literature review, conceptual framework, and theoretical section.** • I find the paper lacking a robust literature review, conceptual or theoretical section (this could focus on 1. global and regional trends in urbanization, especially in developing countries, highlighting how urban expansion affects peri-urban lands and communities, 2. theories related to livelihood diversification and compensation, such as the Sustainable Livelihood Framework, to understand the strategies households employ in response to land loss, 3. Review empirical studies that document the social, economic, and psychological effects of land expropriation on peri-urban farmers, focusing on both challenges and adaptive strategies. 4. Analyzing the specific legal and policy frameworks governing land expropriation in Ethiopia, noting recent changes and their implications for land compensation and livelihood restoration. 5. Identifying gaps in existing literature, particularly concerning long-term impacts on livelihoods and the effectiveness of compensation measures, justifying the need for the current study’s focus.

Response: We have included the literature review to provide a more robust discussion of global and regional trends in urbanization, livelihood diversification theories and the effects of land expropriation.

**Comment 5:** The sample size and sampling technique are well-explained, but the paper could benefit from a more detailed discussion on the limitations of the chosen methods, especially concerning potential biases in key informant interviews and focus group discussions.

Response: We have added a discussion of the limitations of the sampling technique and potential biases in key informant interviews and focus group discussions, as suggested.

**Comment 6:** Consider discussing the validation of the structured questionnaires used for collecting quantitative data to enhance credibility.

Response: We have added a section discussing the validation process of the structured questionnaires used to collect the quantitative data.

**Comment 7:** While the use of binary logistic regression is appropriate, ensure the model assumptions and the choice of variables are justified in more detail. This can include discussing why certain variables were included or excluded and the potential impact of this on the findings

Response: We have expanded the justification for using binary logistic regression, including a discussion of model assumptions and the selection of variables.

**Comment 8:** Provide a more critical analysis of the quantitative data. For instance, discussing the implications of high inflation and lack of urban living skills on livelihood diversification could add depth.

Response: We have included a more detailed analysis of the quantitative data, particularly focusing on how factors such as high inflation and lack of urban living skills influence livelihood diversification strategies.

**Comment 9:** Results are comprehensive but could be better organized to lead the reader through the findings more clearly. Consider using subheadings to distinguish between different aspects of the results, such as economic impacts versus social impacts.

Response: We have reorganized the results section with subheadings to improve clarity, as suggested.

**Comment 10:** Compare the results with similar studies in other regions or contexts, highlighting unique findings and common trends., Based on the findings, recommend specific policy changes or interventions that could improve compensation practices and support livelihood diversification for affected farmers., Discuss practical measures that local governments and development agencies can implement to mitigate the negative impacts of land expropriation on peri-urban farmers.

Response: We have tried to comparing the findings of the study to theoretical expectations and similar studies. We also discuss practical policy recommendations to improve compensation practices and support livelihood diversification for affected farmers.

Comment 11: The policy implications are practical; however, they could be strengthened by suggesting specific policy measures or programs, detailing how they could be implemented within the current Ethiopian administrative and legal framework.

Response: We have revised the policy implications according to your suggestions.

**Comment 12:** Consider adding future research directions, suggesting specific areas that need further investigation based on the study's findings.

Response: We have added a section on future research directions, highlighting specific areas that need further investigation based on the study’s findings.

**Comment 13**: Proof read the paper

Response: Thank you for your suggestion. We have carefully proofread the manuscript to correct any typographical and grammatical errors. Additionally, we have ensured that the manuscript flows more clearly and consistently.
