# Supplementary material for: Effects of urbanization and land expropriation on the livelihoods of peri-urban farmers in North Wollo Zone, Ethiopia
Source: PLoS One. 2025 Jun 26;20(6):e0325985. doi: 10.1371/journal.pone.0325985 (PMC12200778; doi:10.1371/journal.pone.0325985)
Supplement: Attachment — Submitted filename: PLOS-woldia_2.docx [file pone.0325985.s003.docx]

**General Evaluation**

Despite some improvements, the manuscript still lacks sufficient theoretical grounding, conceptual clarity, and integration with broader urbanization and livelihood transformation literature. The study remains largely **descriptive**, with **insufficient analytical depth** in interpreting empirical findings.

### ****Key Issues****

**1. Misuse of the "Mixed-Methods" Label**

The manuscript claims to use a **mixed-methods** approach, yet:

- The **qualitative component is minimal** and superficially integrated.
- There is no detailed thematic presentation or deep narrative analysis from interviews or FGDs.
- The qualitative findings are only used to validate quantitative results, not to enrich or challenge them—making the “mixed-methods” claim questionable.

**Recommendation**: Either significantly strengthen the qualitative section (e.g., coding themes, integrating narratives) or revise the paper to clearly present it as a **quantitative-dominant study** with **supportive qualitative insights**.

**2. Research design:**

- The **actual execution** of the qualitative component in the manuscript **does not meet the expectations** of an explanatory sequential mixed-methods design:
  - **Qualitative data are minimal** (just a few quotes or summary comments from FGDs and KIIs).
  - There is **no structured qualitative analysis** (e.g., coding themes, presenting participant narratives, triangulating).
  - The qualitative findings are **not used to meaningfully explain or reinterpret** the quantitative findings.

**3. Lack of a Clearly Articulated Research Gap**

The introduction claims that the study “addresses several gaps in previous research” but fails to **clearly define or articulate these gaps**. It mostly reiterates findings from earlier studies and then claims that North Wollo Zone is understudied.

- No concrete **theoretical or conceptual** contribution is identified.
- The **"gap" is geographical** rather than scientific or methodological.
- The literature review is descriptive but **not analytical or critical**.

**Recommendation**:

- Clearly **identify what is missing** in existing research (e.g., lack of livelihood transition models, weak compensation policy impact analysis).
- Explain how this study **adds new conceptual or empirical knowledge** rather than just replicating existing frameworks in a new location.

#### 4. **Weak Conceptual Framework**

- While a figure is presented, it is **poorly explained** in the text. The conceptual framework lacks connection to **widely accepted livelihood frameworks** such as the **Sustainable Livelihoods Framework (SLF)**.
- There is **no clear articulation** of how urbanization, expropriation, and compensation relate causally to specific livelihood outcomes beyond descriptive mentions.

**Recommendation**: Embed the study within a recognized theoretical model (e.g., Ellis 2000, DFID 1999 SLF) and elaborate the conceptual linkages in text.

#### 5. **Limited Literature Integration**

- The manuscript reads like a literature summary rather than a critical review. Many statements cite multiple sources but **lack synthesis or critical engagement**.
- International comparisons are limited; much of the cited literature is either Ethiopian or generic, missing insights from broader urban displacement and resettlement research.

**Recommendation**: Engage with recent, comparative urban land studies to better situate findings.

**6. Poorly Constructed Study Area Maps**

The maps are substandard. This remains true:

- The **map of Ethiopia does not label all regions**.
- The **Amhara map lacks administrative zones**.
- The **North Wollo map does not clearly mark the selected towns or woredas**.

**Recommendation**:

- Include **three clearly labeled maps**:
  1. Ethiopia (showing all regions).
  2. Amhara region (with all administrative zones).
  3. North Wollo Zone (clearly highlighting study towns like Woldia, Kobo, Lalibela, Mersa).
- Use proper **map scale, legends, north arrows**, and **standard cartographic conventions**.

#### 7. **Underdeveloped Discussion Section**

- The discussion lacks comparative insight and fails to clearly link results to research questions or broader debates on livelihood resilience or governance.
- Assertions about informality, policy failure, and livelihood decline are not substantiated with in-depth policy or institutional analysis.

**Recommendation**: Expand discussion by linking results to key debates—e.g., expropriation justice, participatory urban governance, resilience of peri-urban communities.

#### **8**. **Conclusion and Policy Implications Are Vague**

- The conclusion restates findings without advancing **clear, actionable policy recommendations**.
- It does not reflect on **institutional responsibilities**, nor does it address **long-term adaptation strategies or urban policy reforms**.

**Recommendation**: Provide **specific, evidence-based policy recommendations**, ideally tiered (e.g., local, regional, national) and connected to institutional gaps found in the study.
