# Supplementary material for: Effects of urbanization and land expropriation on the livelihoods of peri-urban farmers in North Wollo Zone, Ethiopia
Source: PLoS One. 2025 Jun 26;20(6):e0325985. doi: 10.1371/journal.pone.0325985 (PMC12200778; doi:10.1371/journal.pone.0325985)
Supplement: Attachment — Submitted filename: Response_to_Reviewers_auresp_2.docx [file pone.0325985.s005.docx]

**Response to Reviewers**

**Manuscript PONE-D-25-01172R1**

**Title**: Effects of Urbanization and Land Expropriation on the Livelihoods of Peri-Urban Farmers in North Wollo Zone, Ethiopia: A Mixed-Methods Analysis

We would like to thank the editor and reviewers for their valuable feedback and suggestions on our manuscript. We have carefully revised the manuscript in response to the comments provided, and we believe these revisions have substantially improved the quality of the paper. Below, we outline our responses to each of the reviewers' comments.

**General Responses:**

We appreciate the positive feedback regarding the relevance of our study and its focus on urban expansion and land expropriation in Ethiopia, especially in peri-urban areas. We have made significant revisions to address the concerns raised by the reviewers.

**Editor comment #:**

**Comment 1:** Please pay close attention and address the comments by the reviewer.

Response: Thank you for your comment. We have revised our manuscript based on the suggestions of the reviewers and all the comments are corrected.

Comment 2: We note that Figure 2 in your submission contain [map/satellite] images which may be copyrighted. All PLOS content is published under the Creative Commons Attribution License (CC BY 4.0), which means that the manuscript, images, and Supporting Information files will be freely available online, and any third party is permitted to access, download, copy, distribute, and use these materials in any way, even commercially, with proper attribution. For these reasons, we cannot publish previously copyrighted maps or satellite images created using proprietary data, such as Google software (Google Maps, Street View, and Earth). For more information, see our copyright guidelines: http://journals.plos.org/plosone/s/licenses-and-copyright. We require you to either (1) present written permission from the copyright holder to publish these figures specifically under the CC BY 4.0 license, or (2) remove the figures from your submission:

Response: Thank you for your comment.

In response to your concern regarding Figure 2, I have removed the figure from the revised submission due to potential copyright issues, as it contained satellite/map imagery that may not comply with the CC BY 4.0 license requirements. I understand and fully support the journal’s commitment to open access and proper licensing standards.

**Reviewer #1 comments:**

Despite some improvements, the manuscript still lacks sufficient theoretical grounding, conceptual clarity, and integration with broader urbanization and livelihood transformation literature. The study remains largely descriptive, with insufficient analytical depth in interpreting empirical findings.
Key Issues
1. Misuse of the "Mixed-Methods" Label
The manuscript claims to use a mixed-methods approach, yet:
• The qualitative component is minimal and superficially integrated.
• There is no detailed thematic presentation or deep narrative analysis from interviews or FGDs.
• The qualitative findings are only used to validate quantitative results, not to enrich or challenge them—making the “mixed-methods” claim questionable.
Recommendation: Either significantly strengthen the qualitative section (e.g., coding themes, integrating narratives) or revise the paper to clearly present it as a quantitative-dominant study with supportive qualitative insights.

Response: Thank you for this valuable comment. We agreed with your recommendations and we have revised the title to clearly present it as a quantitative-dominant study with supportive qualitative insights.

2. Research design:
• The actual execution of the qualitative component in the manuscript does not meet the expectations of an explanatory sequential mixed-methods design:
o Qualitative data are minimal (just a few quotes or summary comments from FGDs and KIIs).
o There is no structured qualitative analysis (e.g., coding themes, presenting participant narratives, triangulating).
o The qualitative findings are not used to meaningfully explain or reinterpret the quantitative findings.

Response: Thank you for this comment. We have tried to revise this section in this version and consistent with the previous comments.
3. Lack of a Clearly Articulated Research Gap
The introduction claims that the study “addresses several gaps in previous research” but fails to clearly define or articulate these gaps. It mostly reiterates findings from earlier studies and then claims that North Wollo Zone is understudied.
• No concrete theoretical or conceptual contribution is identified.
• The "gap" is geographical rather than scientific or methodological.
• The literature review is descriptive but not analytical or critical.
Recommendation:
• Clearly identify what is missing in existing research (e.g., lack of livelihood transition models, weak compensation policy impact analysis).
• Explain how this study adds new conceptual or empirical knowledge rather than just replicating existing frameworks in a new location.

Response: Thank you for this comment. We have added empirical and methodological gaps in addition to the geographic gap in this version.

4. Weak Conceptual Framework
• While a figure is presented, it is poorly explained in the text. The conceptual framework lacks connection to widely accepted livelihood frameworks such as the Sustainable Livelihoods Framework (SLF).
• There is no clear articulation of how urbanization, expropriation, and compensation relate causally to specific livelihood outcomes beyond descriptive mentions.
Recommendation: Embed the study within a recognized theoretical model (e.g., Ellis 2000, DFID 1999 SLF) and elaborate the conceptual linkages in text.

Response: Thank you for this comment. We have improved the conceptual framework very well and supported by the SLF models. The framework is now thoroughly explained in the text.
5. Limited Literature Integration
• The manuscript reads like a literature summary rather than a critical review. Many statements cite multiple sources but lack synthesis or critical engagement.
• International comparisons are limited; much of the cited literature is either Ethiopian or generic, missing insights from broader urban displacement and resettlement research.
Recommendation: Engage with recent, comparative urban land studies to better situate findings.

Response: Thank you for this comment. The Literature Review has been revised to critically engage with recent comparative literature on urban land governance, displacement, and livelihood transitions from Sub-Saharan Africa, South Asia, and Latin America. We now discuss relevant global studies (e.g., China, India, Bangladesh) to strengthen international comparison and highlight commonalities and differences in peri-urban livelihood disruption.
6. Poorly Constructed Study Area Maps
The maps are substandard. This remains true:
• The map of Ethiopia does not label all regions.
• The Amhara map lacks administrative zones.
• The North Wollo map does not clearly mark the selected towns or woredas.
Recommendation:
• Include three clearly labeled maps:
1. Ethiopia (showing all regions).
2. Amhara region (with all administrative zones).
3. North Wollo Zone (clearly highlighting study towns like Woldia, Kobo, Lalibela, Mersa).
• Use proper map scale, legends, north arrows, and standard cartographic conventions.

Response: Thank you for this comment. We have tried to redesign and included a high-quality maps in version 2. But, I have removed the figure from the revised submission due to potential copyright issues, as it contained satellite/map imagery that may not comply with the CC BY 4.0 license requirements as commented by the editors’.

**Fig 2: Map of the study area**

7. Underdeveloped Discussion Section
• The discussion lacks comparative insight and fails to clearly link results to research questions or broader debates on livelihood resilience or governance.
• Assertions about informality, policy failure, and livelihood decline are not substantiated with in-depth policy or institutional analysis.
Recommendation: Expand discussion by linking results to key debates—e.g., expropriation justice, participatory urban governance, resilience of peri-urban communities.

Response: Thank you for this comment. The Discussion has been thoroughly expanded. We now interpret the findings in the context of linking/comparing the findings to the theoretical framework and broader debates in urbanization, land rights, and rural-urban transitions.

8. Conclusion and Policy Implications Are Vague
• The conclusion restates findings without advancing clear, actionable policy recommendations.
• It does not reflect on institutional responsibilities, nor does it address long-term adaptation strategies or urban policy reforms.
Recommendation: Provide specific, evidence-based policy recommendations, ideally tiered (e.g., local, regional, national) and connected to institutional gaps found in the study.

Response: Thank you for this comment. The Conclusion and Policy Implications section has been rewritten to provide clear, evidence-based, and tiered recommendations at local, regional and national levels.

Reviewer #2: The authors significantly improved the paper. I comment the effort they made. I recommend publication of the paper.

Response: Thank you for your encouragement and support, we really appreciate it.
